# REM-Sleep Behavior Disorder in Patients With Essential Tremor: What Is Its Clinical Significance?

**DOI:** 10.3389/fneur.2019.00315

**Published:** 2019-04-24

**Authors:** Maria Salsone, Gennarina Arabia, Lucia Manfredini, Andrea Quattrone, Carmelina Chiriaco, Basilio Vescio, Miriam Sturniolo, Maurizio Morelli, Rita Nistico', Fabiana Novellino, Antonio Gambardella, Aldo Quattrone

**Affiliations:** ^1^Neuroimaging Research Unit, Institute of Molecular Bioimaging and Physiology, National Research Council, Catanzaro, Italy; ^2^Institute of Neurology, University Magna Graecia, Catanzaro, Italy; ^3^Biotecnomed S.C. aR.L., Catanzaro, Italy; ^4^Neuroscience Center, University Magna Graecia, Catanzaro, Italy

**Keywords:** essential tremor, REM sleep behavior disorder, cognitive impairment, cardiac MIBG scintigraphy, DAT-SPECT imaging

## Abstract

**Objective:** REM sleep behavior disorder (RBD) is an important risk factor for the dementia development and for the deterioration of autonomic functions in patients with Parkinson's Disease. RBD has also been reported in patients with Essential Tremor (ET). However, its clinical significance in ET remains still unknown. We aimed to investigate clinical, neuropsychological and cardiac autonomic scintigraphic differences between ET patients with and without RBD.

**Methods:** To assess RBD symptoms, RBD Single-Question has been administered in a cohort of 55 patients with a clinical diagnosis of ET. Patients with clinical RBD underwent polysomnography (PSG) confirmation. All patients completed a battery of neuropsychological assessment of memory, executive function, attention, language, and visuospatial function. Cardiac MIBG scintigraphy was performed in order to measure the cardiac autonomic innervation.

**Results:** Ten ET patients (18%) had a PSG-confirmed RBD (ET^RBD+^). Compared to ET patients without RBD (ET^RBD−^), significantly reduced scores on memory domain tests such as Rey auditory verbal learning test immediate recall (*p* = 0.015) and Rey auditory verbal learning test delayed recall (*p* = 0.004) and phonemic fluency test (*p* = 0.028) were present in ET^RBD+^. By contrast, no other significant clinical difference has emerged from the comparison between two ET groups. Similarly, ET^RBD+^ patients have cardiac MIBG tracer uptake in the normal value range as occurred in those with ET^RBD−^.

**Conclusions:** This study improves the knowledge on clinical significance of RBD symptoms in ET patients. Our preliminary findings demonstrate that presence of RBD in ET is associated with neurocognitive impairment, but not with cardiac autonomic dysfunction. Further longitudinal studies are needed to investigate whether ET patients with RBD will develop a frank dementia over the time.

## Introduction

Essential tremor (ET) is one of the most common neurological disease among adults. Traditionally, it is defined by a core of clinical motor symptoms characterized by kinetic/postural tremor affecting hand, head, or other parts of the body without other clinical signs of parkinsonism (1). ET, however, is a phenotypically heterogeneous disease including both motor and non-motor symptoms (NMS). In recent years, a growing body of literature has been focused on the prevalence of some of the NMS in ET, such as cognitive impairment, depression, olfactory deficits and sleep disturbances as REM sleep behavior disorder (RBD) (2). Among the NMS, depression and RBD are reported to have higher prevalence in ET patients than in the general population (3).

Interestingly, the NMS especially RBD, found in patients with ET are known to be prodromal conversion symptoms of α-Synuclepathaties such as Parkinson's Disease (PD). However, the presence of RBD in PD patients identifies a specific clinical subtype of the disease. Indeed, in PD, RBD is associated with older age, longer disease duration (4), rigid-akinetic form of PD and more severe parkinsonian symptoms (5). These patients may also have increased autonomic dysfunction and higher risk to develop dementia and therefore worse prognosis (6). Moreover, in PD-RBD patients, cardiac Meta-iodobenzylguanidine (MIBG) uptake, a measure of cardiac autonomic innervation was lower compared to that observed in PD patients without RBD (7).

As occurs in PD, the presence of RBD in ET could identify a specific clinical phenotype. However, the literature regarding the clinical, neuropsychological and scintigraphic features in ET patients associated with RBD is poor or absent. Indeed, only a study (8) has assessed the difference regarding demographics tremor characteristics, and prevalence of autonomic symptoms between ET patients with and without RBD. These authors found that ET patients with RBD had higher scores on Scales for outcomes in Parkinson's Disease-Autonomic Questionnaire (SCOPA-AUT) than those without RBD suggesting that RBD in ET is associated with dysautonomic symptoms. Few reports have investigated cardiac MIBG uptake in patients with ET (9, 10) a no evidence has been reported in ET patients associated with RBD. Finally, no study has previously evaluated neurocognitive performance in ET patients associated RBD.

Thus, several questions regarding the clinical significance of RBD in ET patients are still answered.

Considering that RBD is an important risk factor for the dementia development and for the deterioration of autonomic functions, we aimed to investigate clinical, neuropsychological, and cardiac autonomic scintigraphic differences between ET patients with and without RBD.

## Methods

### Study Population

This cross sectional study included 55 consecutive patients with a clinical diagnosis of ET made by a movement disorders specialist (MS) according to established criteria (11). Each patient underwent an accurate clinical history and a neurological evaluation. Fahn-Tolosa was used for clinical evaluation of ET patients. We assess the presence of clinical symptoms suggestive of REM-sleep behavior disorder (RBD) by using of RBD Single-Question (RBD1Q), a single “yes-no” question querying the classic dream-enactment behavior of RBD (12). According to RBD1Q results, we divided the ET patients into two groups, ET with RBD (ET^RBD+^) and ET without RBD (ET^RBD−^). ET^RBD+^ underwent polysomnographic (PSG) recording. Patients were diagnosed with RBD using polysomnography according to the International Classification of Sleep Disorders, version 3 (ICSD-3) criteria (13). The following cognitive functions were assessed in all enrolled ET patients: (i) global cognitive status (Mini Mental State Examination [MMSE](14); ii) executive functions (Frontal Assessment Battery [FAB] (15), Modified Card Sorting Test [MCST] (16); iii) attention (Digit Span Forward) (17); (iv) verbal short and long term memory, episodic memory (Rey Auditory-Verbal Learning Test Immediate [RAVLT-I] and Delayed [RAVLT-D] (18); v) visuo-spatial functions (Judgments of Line Orientation test form V [JLO-V])(19); (vi) phonemic verbal fluency (Controlled Oral Word Association Test [COWAT] (20); vii) language comprehension [Token Test] (21); (viii) anxiety and depression [Hamilton Rating Scale Anxiety [HRS-A] (22) and Beck Depression Inventory II [BDI-II] (23), respectively]. Before inclusion in the study, written informed consent was obtained from all participants and the study was approved by the institutional review board according to the Helsinki Declaration.

### Imaging Protocol

Imaging protocol included brain MRI, DAT-SPECT, and cardiac MIBG scintigaphy. Participants underwent MRI on a 3T GE system (GE Healthcare, Rahway, NJ). The MRI protocol included: 3-dimensional T1-weighted volumetric spoiled gradient echo (GE), T2-weighted fast spin echo, and T2-weighted fluid attenuated inversion recovery sequences. In all ET patients (with and without RBD) we performed DAT-SPECT (24) to support the clinical diagnosis of ET and Cardiac MIBG scintigraphy to measure the cardiac autonomic innervation thus investigating cardiac autonomic function (24).

#### Cardiac MIBG Scintigraphy

Cardiac MIBG scintigraphy was performed at rest. A total of 111 MBq of I-MIBG (Amersham, Eindhoven, NL) was injected intravenously in 60 s. Data were collected using a dual-head gamma camera (Axis, Picker, Bedford, OH) at 10 min (early image) and 240 min (delayed image) after the isotope injection. Static planar imaging and regional MIBG uptake were obtained with 128 matrix. Only planar images in thoracic anterior view were used for quantitative evaluation. Regions of interest (ROI) were drawn around the whole heart and mediastinum of the anterior image, and tracer uptake was measured within each ROI to calculate the heart/mediastinum (H/M) ratio.

The H/M ratio from early and delayed images was evaluated in all subjects, and values were considered abnormal if they were more than three standard deviations (SDs) below the respective control mean. Regional MIBG uptake was assessed using single-photon emission tomography (SPECT) on the three axes displayed (short axis, vertical long axis, and horizontal long axis). Images were evaluated by an investigator who was blinded to the patients' diagnosis (24).

### Statistical Analysis

Differences in distribution of sex, familiarity, and clinical features between ET^RBD+^ and ET^RBD−^ groups were assessed by means of the Fisher's exact test. The Shapiro-Wilk test was used to check for normality before performing comparisons between continuous variables. When comparing ET^RBD+^ and ET^RBD−^ groups, the Mann–Whitney *U*-test was used to assess differences in age at evaluation, education, disease duration, age at ET onset, Fahn-Tolosa score, part A and part B of Fahn-Tolosa scale, MMSE, MCST, JLO-V, Digit Span scores, HRS-A and BDI-II, while DAT-SPECT, MIBG, Token test, RAVLT I.R., RAVLT D.R., FAB, and phonemic fluency test scores were compared by means of Student's *t*-test. In order to control for false discovery rate, Benjamini–Hochberg correction was applied to *p*-values when comparing neuropsychological variables. All tests were two-tailed and the α level was set at *p* < 0.05. Statistical analysis was performed with R Statistical Software (R for Unix/Linux, version 3.1.1, The R Foundation for Statistical Computing, 2014).

## Results

### Demographics and Clinical Characteristics

According to RBD1Q results, 10 ET patients (18%) were positive (ET^RBD+^) whereas 45 were negative (ET^RBD−^). All ET^RBD+^ patients received a PSG-confirmation of clinical suspicion of RBD. Sleep and dream-related behaviors reported by the history and documented during video PSG were present in our ET^RBD+^ patients and included violent complex motor behaviors both disruptive (60%) to the bed partner (punching, kicking etc.) and injurious (40%) (biting an arm, leaping from the bed etc.). Indeed, ET^RBD+^ patients showed clear abnormal REM sleep behaviors during PSG recording. Interestingly, in three patients with ET^RBD+^, RBD preceded the onset of motor symptoms by several years while in 1 patient was contemporary. Demographic and clinical characteristics of all participants are summarized in Table 1. Patients groups were not statistically different regarding sex, onset, family history, disease duration, and severity of disease. Patients with ET^RBD+^ had a slight higher prevalence of head tremor, kinetic tremor, and lower of asymmetrical postural tremor than those with ET^RBD−^. NMS such as hyposmia and constipation did not show significant difference between two groups (Table 1).

**Table 1:** Comparisons among demographic, clinical, and neuropsychological data of patients affected by ET, ET^RBD+^, and ET^RBD−^.

**Variables**	**All ET group (*N* = 55)**	**ET^**RBD+**^ (*N* = 10)**	**ET^**RBD–**^ (*N* = 45)**	***p*-value**
**DEMOGRAPHICS**				
Sex: No. men/women	25/30	7/3	21/24	0.75^a^
Age, years (mean ± SD)	65.0 ± 10.1	62.9 ± 12.2	65.5 ± 9.6	0.52^b^
Education, years (mean ± SD)	9.4 ± 4.5	10.8 ± 2.5	9.1 ± 4.8	0.12^b^
**FAMILY HISTORY**				
Postural/kinetic tremor, n. (%)	30 (54.5)	5 (50)	25 (56.8)	0.74^a^
**DISEASE FEATURES**				
Disease duration, years (mean ± SD)	13.9 ± 14.3	10.1 ± 9.2	14.7 ± 15.7	1^b^
Age at onset of ET, years (mean ± SD)	51.5 ± 16.3	52.7 ± 13.3	51.3 ± 17.0	0.99^b^
Head tremor, n. (%)	27 (45.4)	6 (60)	19 (42.2)	0.48^a^
Kinetic tremor, n. (%)	41 (74.5)	9 (90)	32 (71.1)	0.1^a^
Asymmetric postural tremor, n. (%)	31 (53.5)	4 (40)	26 (57.8)	0.34^a^
Fahn-Tolosa score (mean ± SD)	22.3 ± 12.9	17.7 ± 7.1	23.8 ± 14.3	0.11^c^
Part A of Fahn-Tolosa scale (mean ± SD)	7.3 ± 3.1	6.7 ± 2.3	7.5 ± 3.4	0.72^b^
Postural tremor, n. (%)	55 (100)	10 (100)	45 (100)	1^a^
Part B of Fahn-Tolosa scale (mean ± SD)	9.4 ± 7.3	8.0 ± 5.2	10.0 ± 8.1	0.39^c^
Hyposmia/Anosmia, n. (%)	4 (7.2)	1 (10)	3 (6.6)	1^a^
Constipation, n. (%)	10 (18.1)	1 (10)	9 (22.5)	0.71^a^
**NEUROPSYCHOLOGICAL BATTERY**				
MMSE mean ± SD (range)	25.7 ± 3.7	26.3 ± 2.5	25.6 ± 3.9	0.78^b^
Token test mean ± SD (range)	30.1 ± 2.5	29.4 ± 2.2	30.2 ± 2.6	0.46^c^
RAVLT I.R. mean ± SD (range)	36.2 ± 10.4	29.0 ± 6.6	37.7 ± 10.5	0.015^c^
RAVLT D.R. mean ± SD (range)	6.9 ± 2.8	4.2 ± 2.0	7.4 ± 2.6	0.004^c^
MCST mean ± SD (range)	4.7 ± 2.2	4.7 ± 2.3	4.7 ± 2.3	0.93^b^
FAB (mean ± SD)	14.1 ± 1.8	13.7 ± 1.2	14.1 ± 1.9	0.55^c^
JLO-V (mean ± SD)	22.2 ± 5.5	21.5 ± 5.6	22.4 ± 5.6	0.76^b^
Digit Span (mean ± SD)	5.3 ± 3.2	4.6 ± 0.2	5.4 ± 3.4	0.50^b^
COWAT (mean ± SD)	24.0 ± 5.9	20.3 ± 3.8	24.7 ± 6.0	0.028^c^
HRS-A (mean ± SD)	10.8 ± 4.5	11.2 ± 4.8	10.6 ± 4.6	0.95^b^
BDI-II (mean ± SD)	11.9 ± 5.9	12.6 ± 6.0	11.6 ± 6.1	0.77^b^

ET^RBD+^ group, Essential tremor (ET) patients with Rem sleep behavior disorder (RBD); ET^RBD−^, ET patients without RBD;

MMSE, Mini Mental State Evaluation (n.v. ≥ 24); Range, ET^RBD+^ group, (25–29); ET^RBD−^ group, (25–30);

Token test (n.v. ≥ 26.25); Range, ET^RBD+^ group, (27–31); ET^RBD−^ group, (27.75–34);

RAVLT R.I., Rey auditory verbal learning test immediate recall (n.v. ≥ 28.53); Range, ET^RBD+^ group, (21.8–33.2); ET^RBD−^ group, (29–67.3);

RAVLT R.D., Rey auditory verbal learning test delayed recall (n.v. ≥ 4.69); Range, ET^RBD+^ group, (1.6–6.9); ET^RBD−^ group, (5–11.8);

MCST, Modified card sorting test (n.v. ≥ 3); Range, ET^RBD+^ group, (3–6); ET^RBD−^ group, (3–6);

FAB, frontal assessment battery (n.v. ≥ 13.4); Range, ET^RBD+^ group, (12.2–14.4); ET^RBD−^ group, (12.9–18);

JLO-V, Judgment of Line Orientation-Form V (n.v. ≥ 20); Range, ET^RBD+^ group, (21–26); ET^RBD−^ group, (21–27);

Digit span (n.v. ≥ 3.5); Range, ET^RBD+^ group, (4.25–4.75); ET^RBD−^ group, (3.75–6.25);

COWAT, Controlled Oral Word Association Test (n.v: ≥ 17.35); Range, ET^RBD+^ group, (17.3–25.5); ET^RBD−^ group, (17.9–38.2);

HRS-A, Hamilton Rating Scale Anxiety (n.v. > 14); Range, ET^RBD+^ group, (8–14); ET^RBD−^ group, (9–18);

BDI-II, Beck Depression Inventory II (n.v: > 13); Range, ET^RBD+^ group, (8–18); ET^RBD−^ group, (8–19);

*n.v., normal values in Italian Population*.

a *Fisher's exact test*.

b *Mann–Whitney U-test (Wilcoxon rank sum test)*.

c *Two-sample t-test. P-values are calculated ET^RBD+^ group vs. ET^RBD−^ group*.

*Multiple comparison tests: RAVLT D.R p = 0.038; RAVLT R I.R., p = 0.068; COWAT, p = 0.084*.

### Neurological Test Scores

The neurological test scores and results of analyses are presented in Table 1. Neuropsychological assessment revealed that ET^RBD+^ had significant lower scores concerning verbal short and long term memory tests such as RAVLT I.R. (*p* = 0.015) and RAVLT D.R. (*p* = 0.004) and phonemic verbal fluency as COWAT (*p* = 0.028) than ET^RBD−^. Moreover, RAVLT D.R results resist multiple comparisons (*p* = 0.038) with a trend for RAVLT I.R. (*p* = 0.068) and COWAT (*p* = 0.084). Of note, although ET^RBD+^ patients had slightly higher MMSE scores than ET^RBD−^ patients, they showed overall cognitive performances lower for FAB, MCST, JLO-V, and Token Test compared to those of patients with ET^RBD−^. Finally, concerning anxiety and depression, ET^RBD+^ patients did not significant differ from those with ET^RBD−^ (Table 1).

### Imaging Results

**Figure 1:**
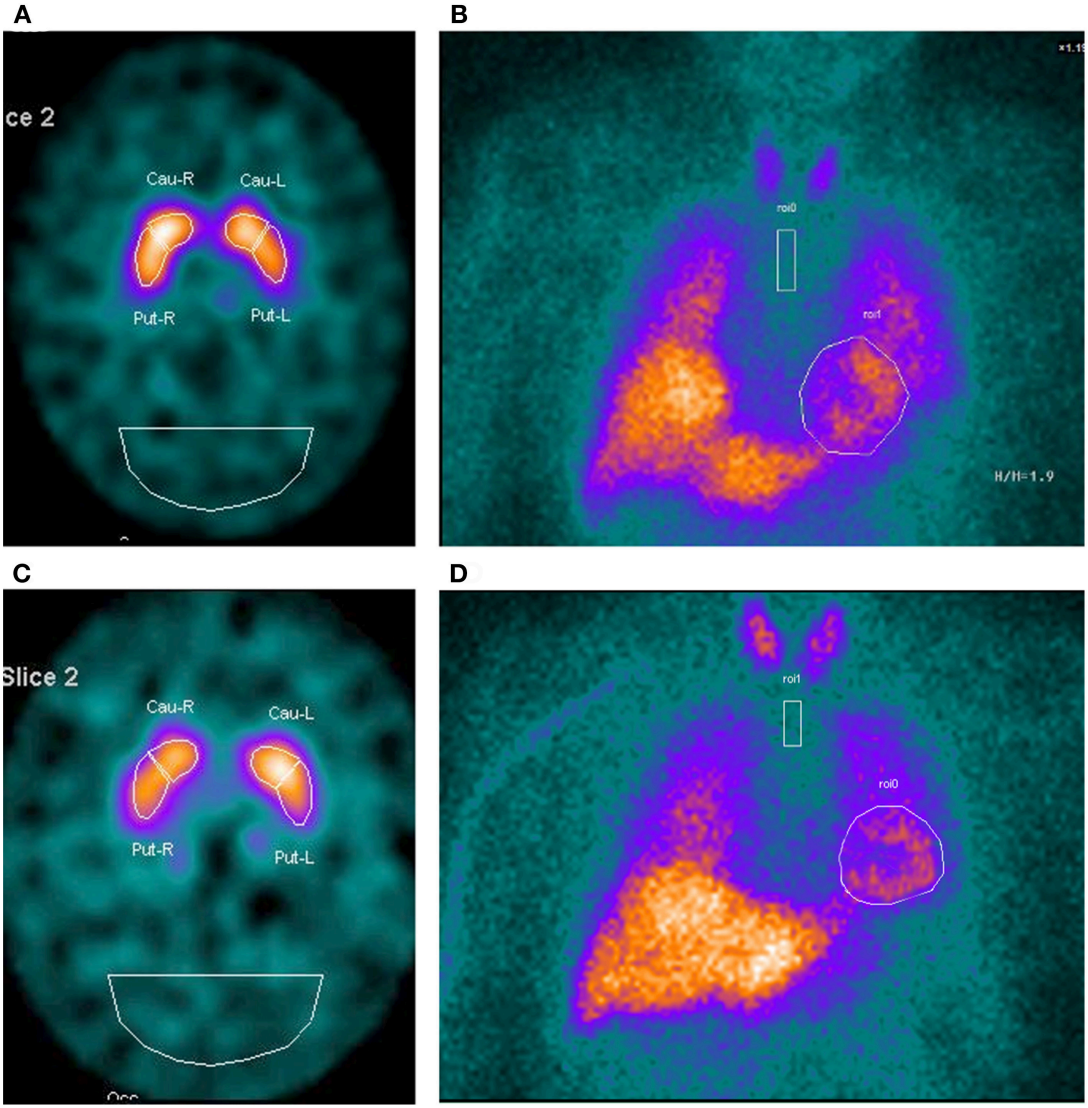
DAT-SPECT imaging and cardiac MIBG scintigraphy in a patient with ET ^RBD+^ **(A,B)** and in a patient with ET^RBD−^ **(C,D)**. The images show in both patients a normal uptake of the tracers.

MRI scan indicated no signal abnormalities in any ET patient. Table 2 shows the comparisons among scintigraphic data of patients affected by ET^RBD+^ and ET^RBD−^. No ET patient had a damage of nigrostriatal presynaptic dopaminergic system on DAT-SPECT imaging thus supporting the clinical diagnosis of ET in both groups. In addition, DAT-SPECT tracer uptake did not differ in qualitative and quantitative (Putamen/Occ ratio) analyses between two groups (Table 2). Similarly, cardiac MIBG uptake (Heart/Mediastinum ratio) both early and delayed images, showed no difference between the two groups (Table 2). Finally, DAT-SPECT and cardiac MIBG uptakes were both within the normal range values in the two groups of ET (Table 2). Figure 1 shows the qualitative images of DAT-SPECT and cardiac MIBG tracers in a patient with ET^RBD+^ (Figures 1A,B) and in patient with ET^RBD−^(Figures 1C,D).

**Table 2:** Comparisons among scintigraphic data of patients affected by ET, ET^RBD+^, and ET^RBD−^.

**Variables**	**All ET group (*N* = 55)**	**ET^**RBD+**^ group (*N* = 10)**	**ET ^**RBD–**^ group (*N* = 45)**	***p*-value^a^**
DAT-SPECT IMAGING^*^				
Putamen R/Occ ratio	4.41 ± 0.61	4.64 ± 0.45	4.36 ± 0.6	0.12^a^
Putamen L/Occ ratio	4.38 ± 0.62	4.62 ± 0.60	4.34 ± 0.61	0.20^a^
CARDIAC MIBG SCINTIGRAPHY^**^				
Heart/Mediastinum ratio early image	1.72 ± 0.25	1.81 ± 0.3	1.70 ± 0.24	0.28^a^
Heart/Mediastinum ratio delayed image	1.73 ± 0.28	1.84 ± 0.33	1.69 ± 0.25	0.21^a^

ET^RBD+^ group, Essential tremor (ET) patients with Rem sleep behavior disorder (RBD); ET^RBD−^, ET patients without RBD; DAT-SPECT, Dopamine transporter ligand (DAT)-Single photon emission computerized tomography (SPECT); Putamen/Occ ratio [Putamen specific (Left/Right) to non-specific (occipital) area]; MIBG, Cardiac I-^123^ Metaiodobenzylguanidine (MIBG) scintigraphy;

* *Normal values: Put/Cau right (mean ± SD, 4.29 ± 0.34) and Put/Cau left (mean ± SD, 4.19 ± 0.39)*.

** *Normal values: Heart/Mediastinum ratio: mean ± SD, 1.94 ± 0.18 early; 2.02 ± 0.19 delayed*.

a *Two-sample t-test. P-values are calculated ET^RBD+^ group vs. ET^RBD−^ group*.

## Discussion

Our goal was to investigate for the first time clinical, neuropsychological, and scintigraphic differences between ET patients with and without RBD. In particular, we found that neurocognitive function, including verbal short and delayed memory and phonemic verbal fluency, was worse in ET patients with RBD than in those without RBD. By contrast, no significant clinical and scintigraphic difference emerged between the two ET groups. Our preliminary findings suggest that ET patients with RBD could be a subgroup of ET at higher risk to develop a frank dementia over the time.

The presence of RBD in ET patients raises some clinical questions. First, RBD and dementia development. Accumulating evidence suggest that RBD is an important determinant of cognitive impairment in patients with α-Synucleinopathies as Parkinson's Disease (PD). Most studies have reported that the prevalence of MCI was significantly higher (until 70%) in PD with RBD than in those without RBD (25). A longitudinal study (26) also found that all PD-RBD with MCI on baseline (48%) developed a frank dementia on 4 years' follow-up evaluation thus suggesting that RBD may be a valid phenoconversion biomarker of dementia.

In our study we questioned whether in ET patients, as occurs in those with PD, RBD could be associated with neurocognitive dysfunctions. We found that all ET patients with PSG-confirmed RBD (18%) (ET^RBD+^) had worse cognitive abilities than those of ET patients without RBD (ET^RBD−^). In particular, although ET^RBD+^ patients had slightly higher MMSE scores, they showed overall executive and visuospatial functions, attention and language comprehension worse than those with ET^RBD−^. Of note, compared to ET^RBD−^ significantly lower performances on RAVLT I.R. (Immediate) and RAVLT D.R. (Delayed) were present in ET^RBD+^. The RAVLT is a powerful neuropsychological tool widely used for the cognitive assessment in dementia and pre-dementia conditions. It is sensitive to verbal memory deficits caused by several neurological diseases (27, 28). Different scores may be derived from RAVLT, but RAVLT Immediate and Delayed are the most used scores in the clinical setting since they highlight different aspects of episodic memory (learning and delayed memory, respectively). Thus, RAVLT is considered an effective marker for discriminating normally aging subjects from MCI and Alzheimer's disease (AD) patients (29). Decreased RAVLT performance found in our ET^RBD+^ could reflect a deficit verbal episodic memory. Of note, in our study RAVLT Delayed resisted multiple comparisons thus suggesting that our results are solid. Interestingly, we also found a significantly lower performance on COWAT in ET^RBD+^ compared to ET^RBD−^. The COWAT allows to evaluate phonemic verbal fluency thus investigating both language and executive function domains. Recent evidence (30) has demonstrated that a decreased COWAT performance is a strong predictor of conversion from normal cognition to preclinical AD. Thus, the decreased score on COWAT found in our ET^RBD+^ could be suggestive of initial mild cognitive impairment. Moreover, we are in agreement with previous evidences (31) reporting that in RBD symptoms usually correlate with specific cognitive domains including verbal memory and executive functions. Supporting this hypothesis, some authors (32) also found that PD-RBD patients performed worse than PD-nRBD in attention, executive functions, verbal learning and memory RAVLT I.R. and RAVLT D.R., thus suggesting that the presence of RBD in their PD patients was associated with increases the risk of a MCI diagnosis. Taken together our evidences, although preliminary, suggest that RBD in ET could be associated with cognitive impairment.

Cognitive impairment and dementia, however, are not surprising in ET. A series of neuropsychological investigations (33–36) have well-documented that ET may exhibit a clinical spectrum of mild cognitive deficits including attention, executive function, memory, and naming. Indeed, has been reported that ET patients have an increased risk for developing both amnestic and non-amnestic MCI (37). Neurocognitive deficits in ET, are usually deficits in specific aspects of neurocognitive functioning particularly those thought to rely on the integrity of the prefrontal cortex suggesting an involvement of fronto-cerebellar circuits (33, 34). Moreover, epidemiological evidences have demonstrated ET patients have greater risk of developing dementia and at a faster rate of progression than in normal elders thus suggesting that this could be not a simple age-related consequence (38–42). It remains still unknown, however, whether ET patients exhibiting cognitive impairment had or not RBD symptoms. In our study, only ET patients with RBD showed cognitive impairment with scores on Immediate, Delayed RAVLT and COWAT similar to those observed in ET patients with cognitive impairment (34). Thus, we can speculate that ET patients with cognitive impairment reported in the latter cited studies, could have clinical or subclinical RBD symptoms.

The second question regarding the presence of RBD in ET is the association with dysautonomic symptoms and the development of α-Synucleinopathies as PD. Some authors (8) reported that ET with RBD had higher prevalence of dysautonomic symptoms compared to those without RBD. As these symptoms are known to be PD prodromal symptoms, they suggest that ET-RBD may be a subgroup of ET at higher risk for PD progression (8). The biological support for this notion could consist in neuropathological investigations revealing the presence of Lewy bodies in some ET brains defining a “Lewy bodies ET subtype” (43). On the other hand, it is well-documented that in idiopathic RBD (iRBD) patients, the initial α-synuclein aggregation targets the nerve terminals of the peripheral autonomic nervous system (44). Thus, we investigated in all ET patients (with and without RBD) the integrity of cardiac autonomic system using cardiac MIBG scintigraphy, a tool able to measure the cardiac autonomic innervation. Indeed, cardiac MIBG scintigraphy has been recently proposed to be a useful predictor of RBD phenoconversion. When iRBD converts vs. Lewy bodies disease as PD, it is characterized by cardiac sympathetic denervation whereas it converts vs. multiple system atrophy, cardiac sympathetic innervation is preserved (44). Only two studies (9, 10) have previously investigated cardiac autonomic innervation in ET. Both studies, found that in ET cardiac sympathetic innervation was preserved unlike to occur in PD. We are in agreement with these evidences since in ET^RBD+^ cardiac MIBG uptake was in the normal value range as occurred in ET^RBD−^. This finding is strongly indicative of preserved cardiac sympathetic innervation and suggest that in our ET cohort, the presence of RBD was not associated with cardiac sympathetic system damage. In addition, there was any clinical significant difference between ET patients with and without RBD concerning, demographics, tremor characteristics (kinetic, postural tremors etc.) and prevalence of other NMS such as constipation and hyposmia. Considering the lack of clinical and imaging differences between ET patients with and without RBD, we suggest that ET patients with RBD could be a subgroup belong to ET syndrome rather than a subgroup higher risk for PD progression. This assertion needs confirmation in future studies.

Our study has some limitations. The most significant is the lack of neuropathological investigation thus we cannot exclude that ET^RBD+^ have Lewy bodies in the brains. Cardiac MIBG scintigraphy, however, is considered a valid tool to investigate the cardiac sympathetic system, a system usually damaged in patients with Lewy bodies disease as PD. Second, the two subgroups of ET patients have different sample sizes, probably caused by the prevalence of RBD in ET. However, in our study we found statistical significant differences between two groups resisting multiple comparison (RAVLT D.R.) thus suggesting that discrepancy does not affect the obtained results. Third, we used RBD1Q to investigate clinical symptoms of RBD and to screen the subjects to send to PSG confirmation. This questionnaire is widely used in clinical setting having a sensitivity of 93.8% and a specificity of 87.2% for identifying subjects with RBD clinical suspicion. Finally, the lack of a follow up period of evaluation. Further longitudinal studies in a wider cohort of ET patients with RBD are needed to investigate whether these patients will develop a frank dementia over the time.

Despite to these limitations, our preliminary results are important to better characterize the clinical phenotype ET-RBD. Reduced performances on verbal short and delayed memory and phonemic verbal fluency test reported in patients with RBD, but not in those without RBD, suggest that ET patients with RBD may be subgroup at higher risk to developing dementia.

## Author Contributions

MaS contributed to the design of the study, drafting and revising the manuscript, analysis, acquisition, and interpretation of the data. GA, MM, RN, FN, and AG contributed to the analysis and the interpretation of the data. LM and AnQ contributed to the acquisition and analysis of the data. BV contributed to statistical analysis of the data. AlQ contributed in the study concept/design, critical supervision of the article, and approved the final version of the manuscript. CC contributed to the acquisition and analysis of the neuropsycological data. MiS contributed to the acquisition of the PSG data.

### Conflict of Interest Statement

The authors declare that the research was conducted in the absence of any commercial or financial relationships that could be construed as a potential conflict of interest.
